# Correction: Luna-Perejón et al. Smart Shoe Insole Based on Polydimethylsiloxane Composite Capacitive Sensors. *Sensors* 2023, *23*, 1298

**DOI:** 10.3390/s25113560

**Published:** 2025-06-05

**Authors:** Francisco Luna-Perejón, Blas Salvador-Domínguez, Fernando Perez-Peña, José María Rodríguez Corral, Elena Escobar-Linero, Arturo Morgado-Estévez

**Affiliations:** 1 E.T.S. Ingeniería Informática, Avda. Reina Mercedes s/n, Universidad de Sevilla, 41012 Seville, Spain; 2 Department of Automation, Electronics and Computer Architecture and Networks, Escuela Superior de Ingeniería, Universidad de Cádiz, Avda. Universidad de Cádiz 10, 11519 Puerto Real, Spain; 3 Department of Computer Science and Engineering, Escuela Superior de Ingeniería, Universidad de Cádiz, Avda. Universidad de Cádiz 10, 11519 Puerto Real, Spain

## Text Correction

There was an error in the original publication [1]. Reviewer 2 recommended an irrelevant reference during peer review.

A correction has been made to Section 2, Paragraph 9. The sentence “These include everything from the development of devices which monitor vital signs [39] to the detection of stress [40] or habits that put health at risk [41].” has been updated to “In the context of body mechanics analysis, this includes applications ranging from stress detection [39] to identifying biomechanical habits that pose a risk to health [40].”.

## References Correction

Reference [39] has been removed. With this correction, the order of some references has been adjusted accordingly.

The authors state that the scientific conclusions are unaffected. This correction was approved by the Academic Editor. The original publication has also been updated.
